# Support after Brain Tumor Means Different Things: Family Caregivers’ Experiences of Support and Relationship Changes

**DOI:** 10.3389/fonc.2015.00033

**Published:** 2015-02-12

**Authors:** Tamara Ownsworth, Elizabeth Goadby, Suzanne Kathleen Chambers

**Affiliations:** ^1^School of Applied Psychology, Griffith Health Institute, Griffith University, Brisbane, QLD, Australia; ^2^Cancer Council Queensland, Brisbane, QLD, Australia; ^3^Health and Wellness Institute, Edith Cowan University, Brisbane, QLD, Australia; ^4^Centre for Clinical Research, University of Queensland, Brisbane, QLD, Australia

**Keywords:** family caregivers, brain tumor, support, relationships, qualitative research

## Abstract

Shorter hospital stays and greater emphasis on outpatient care means that family members have the primary responsibility for supporting a person with brain tumor to manage the physical, cognitive, behavioral, and emotional effects of the illness and its treatment. Given the integral role of family caregivers, it is essential to understand their experience of the impact of brain tumor and their own support needs. Accordingly, this qualitative study aimed to investigate family caregivers’ experiences of support and relationship changes in the context of brain tumor. In-depth interviews were conducted with 11 family caregivers (8 spouse/partner, 3 parents) of people with malignant or benign tumor. A thematic analysis of interview transcripts identified two major themes, namely, “Meanings of Support” and “Relationship Impacts.” The Meanings of Support theme was characterized by intertwined and distinct support needs, varied expectations of support and factors influencing support expectations. The Relationship Impacts theme depicted mixed experiences of strengthened, maintained, and strained relations with the person with brain tumor. Overall, the findings highlight that there is considerable variability in caregivers’ experiences and expectations of support and the impact of brain tumor on relationships. The implications of these findings for the provision of caregiver support are discussed.

## Introduction

Family caregivers of people with brain tumor experience high levels of stress related to unique care demands associated with both cancer and brain injury. Stressors include their loved one’s uncertain prognosis, protracted treatment, and reduced lifespan, as well as neurocognitive deficits and personality changes commonly arising from the tumor or its treatment (1). Due to the trend for shorter hospital stays and increased emphasis on outpatient care, family members assume the primary role in supporting individuals to cope with their symptoms and the day-to-day impact of the illness. Although the majority of primary caregivers are spouses, parents, and children can also function within a support role, thus reinforcing the notion that brain tumor is a family disease (2). There is a paucity of research examining caregivers’ experiences in the context of brain tumor, particularly studies focusing on their own support needs and the impact of brain tumor on relationships.

A diagnosis of a brain tumor is usually traumatic and can occur after the sudden onset of neurological symptoms such as a seizure, or following a prolonged period of more gradual and perplexing changes in a person’s functioning (3). Caregivers often find themselves in a rapidly changing situation with a short time frame between the diagnosis, start of treatment, and their commencement of caregiver responsibilities (4). Most feel under-prepared and overwhelmed by the demands of caregiving, which may vary from minimal assistance with activities of daily living (ADLs) to the complete care and supervision of someone with severe disability (5, 6). Caregiver tasks include supporting the person with basic and instrumental ADLs, monitoring his/her health status and administering medication, organizing and attending appointments, decision making, and providing emotional and social support (7, 8). Caregivers also usually assume greater responsibilities for childcare, running the household, earning an income, and managing finances (3, 9).

The increased responsibilities placed on family members can lead to significant strain on their relationship with the person with brain tumor and, in some cases, relationship breakdown (9). Carlson (10) reported that females with brain tumor were nearly 10 times more likely to become divorced or separated during the course of their illness compared to males with brain tumor. Other negative consequences of caregiving include physical and psychological health problems and economic and social burden (9, 11, 12). Caregivers often perceive their role as physically exhausting and experience health problems such as insomnia and headaches (3, 7). They also commonly develop mental health problems, with 20–30% endorsing clinical levels of depression (13, 14), 40–60% reporting clinical levels of anxiety (13, 14), and 35% found to experience significantly higher levels of stress than the general population (15). In a study by Petruzzi and colleagues (16), caregivers were found to report poorer quality of life than individuals with brain tumor.

The considerable uncertainty associated with the illness represents a major source of stress for caregivers. In a series of in-depth interviews conducted over a 6-month period, Wideheim et al. (17) identified that caregivers of people with malignant brain tumor experienced fear concerning their loved ones’ prognosis for survival and treatment outcome and had a low sense of security in their everyday lives. Planning ahead was difficult and caregivers often wanted to be near their loved one in case their condition deteriorated. Edvardsson and Ahlstrom (7) similarly found that caregivers experienced a sense of uncertainty, shock, despair, and apprehension about the future as the tumor progressed. Caregivers referred to the “emotional rollercoaster” experienced when a set-back (e.g., seizures) occurred, which reminded them of the life threatening nature of brain tumor.

The neurological and functional effects of the illness (e.g., behavioral and personality changes) can restrict the social participation of people with brain tumor and their caregivers and contribute to a sense of isolation (11, 17, 18). In particular, Edvardsson and Ahlstrom (7) identified that caregivers often felt “invisible and neglected” by friends, family and doctors. Loss of friends and diminished social ties contributed to caregivers’ grief regarding the long-term prospects of caring for an individual with extensive care needs without the benefit of social support (11). Consistent with this notion, research has identified that social support can buffer the impact of functional impairments. Specifically, caregivers supporting an individual with more severe functional impairment had better psychological wellbeing when they were highly satisfied with their social support (19).

Research has found that caregivers especially value support from family and friends during the early phases of diagnosis and treatment (11, 19, 20). Interestingly, Hricik et al. (21) found that as the disease progressed, caregivers often sought more support from people going through a similar situation because they were able to relate to their situation and provide information on how to cope. Brain tumor support groups and online support networks can provide a helpful forum for caregivers to troubleshoot difficult situations and express their frustration. Support groups can also serve as a valuable source of information and help caregivers to maintain their morale (3, 11). However, it can be difficult for caregivers to access support for their own needs because the focus of support is generally on the person with brain tumor (7).

Caregivers of people with brain tumor perceive a range of unmet support needs, including a lack of practical support, such as help managing financial issues and government agencies, access to information about brain tumor and caregiving (5, 22) and existential and emotional support around end of life issues (5, 9, 11, 12, 23, 24). Cornwell and colleagues (23) found that caregivers were often unsure about support available to them, and expressed that they would have accessed services, such as support groups, if they had been made aware of them by hospital staff. Janda and colleagues (11) identified some parallels in the support needs of people with brain tumor and their caregivers. Although many of the unmet practical, informational, and emotional support needs were similar, their study did not specifically investigate caregivers’ support needs as distinct from the needs of the person with brain tumor. Furthermore, the influence of support on caregivers’ ability to adjust to their changing roles in the family was not explored.

Changes in relationship dynamics and family roles have been highlighted in numerous brain tumor studies (3, 9, 25, 26). In particular, caregivers have described their experience of grieving the loss of the person still living and a loss of intimacy and relationship breakdown (1, 9). McConigley et al. (4) referred to the process of “renegotiating relationships,” which was required to adapt to changes, such as the person with brain tumor no longer being able to contribute intellectually or financially to the relationship due to cognitive difficulties. Spousal caregivers often perceived a loss of equality in their relationship whereby they no longer had an equal partnership (4, 27). In the study by Edvardsson and Ahlstrom (7), some caregivers described feeling like a single parent, despite being one of two parents. They also perceived a change from a being romantic partner to assuming the role of parent due to helping the individual with personal care tasks such as dressing and hygiene. Such role changes were distressing for some caregivers, whereas others viewed these in more positive light, expressing their satisfaction with supporting their loved one with these tasks (7). Salander (28) found that personality changes were the hardest to adjust to. In particular, spousal caregivers were more likely to report relationship strain when their spouse displayed personality changes (e.g., “demanding” and “dominating”), which contributed to caregivers distancing themselves from the person.

Notwithstanding the detrimental physical, psychological, and social effects, caregivers have also been found to report positive outcomes associated with their role. Consistent with findings in the broader cancer literature (8, 29), providing care to a person with brain tumor can have many positive psychological consequences, including increased strength and resilience, greater appreciation of life and development of closer relationships (7, 27, 30, 31). For example, some caregivers of people with primary malignant brain tumor felt they had formed a stronger bond with their loved one because the illness created more opportunities to spend time together (27). In their interviews of bereaved caregivers, Sherwood et al. (3) identified that many caregivers felt “grateful” and “privileged” to have provided care to the person with brain tumor and perceived a strengthening of their relationship. In reflecting on the past, caregivers identified both difficult and satisfying aspects of their role. Although it is evident that caregiving can be associated with negative and positive consequences for relationships, the influence of support on relationship changes is unclear.

Overall, there is little understanding of caregivers’ perceptions of their support needs and how these may differ to those of the person with brain tumor. Given the findings that lack of social support contributes to psychological distress and lower perceptions of coping (19, 23), greater understanding of caregivers’ perception of their own support needs is essential to provide holistic psychosocial support. Further, although changes in family roles and responsibilities after brain tumor have been well researched, the issues contributing to relationship changes and the influence of support has received little attention.

Qualitative research methods are particularly well suited to understanding complex social situations or contexts in which the perceptions of the people directly involved provide a rich source of data (32). The aims of this qualitative study were, first, to investigate how caregivers perceive their support needs, and second, to identify relationship changes in the context of brain tumor. The two main research questions were as follows:

1. How do caregivers perceive their support needs in the context of brain tumor? In addressing this question, emphasis was placed on their perceptions of (a) the support needs of the person with brain tumor; and (b) the caregiver’s own support needs.
2. How does brain tumor impact on the relationship between the caregiver and person with brain tumor? Additionally, the influence of social support on relationship changes was explored.

## Materials and Methods

### Design

The study methodology was informed by guidelines for conducting and assessing qualitative research, as summarized in Table 1 (33–35). A phenomenological approach was considered most consistent with the nature of the aims and research questions. Phenomenology is concerned with understanding “the meaning, structure, and essence of the lived experience of this phenomenon, for this person or group of people” [(36), p. 104]. Interviews are the most common means of data collection and data analysis techniques are designed to facilitate the interpretation of meaning (37). Questionnaire data were also used in the current study to provide information about caregivers’ psychological functioning and their sources of and satisfaction with social support.

**Table 1:** **Guidelines and considerations for conducting and appraising qualitative research [see Ref. (33–35)]**.

Guidelines	Specific considerations
Relevance of research	Research question is relevant
	Aim is sufficiently focused and clearly stated
Appropriate method and design	Qualitative research method chosen is the best approach for the research question/aims
	Researchers acknowledge their personal background and experiences relevant to the phenomenon under investigation (i.e., reflexivity)
Data collection and sampling	Strategy for data collection is clearly stated and appropriate to the research question
	Theoretical: based on preconceived or emergent theory
	Purposive: diversity of opinion
	Volunteer: feasibility, hard to reach groups
	Justification for the approach is given
	Recruitment is conducted using appropriate methods
	Characteristics of the sample and setting are stated clearly and in sufficient detail
Data analysis	The type of analysis is appropriate for the study
	Principles and procedures for data analysis are fully described
	How categories and frameworks were identified is clearly stated
	Trustworthiness/rigor of the data and interpretation is established (e.g., triangulation)
Findings	Quotes are used appropriately and effectively to support findings
	Findings are relevant to the aims
Discussion	Findings are compared with appropriate theoretical and empirical references
	The design is scrutinized
	Limitations are considered
	Clear consequences of the study are proposed
Ethical issues	Approval from an appropriate ethics committee received
	Informed consent was sought and granted
	Participants anonymity and confidentiality ensured
Clarity	Well-written and accessible

### Participants

Caregiver participants (*n* = 25) were part of a broader study, which examined how individuals with brain tumor make sense of and adjust to their illness (38). In this broader study, individuals with brain tumor (*n* = 30) were recruited from a brain tumor support group or a neurosurgical practice and interviewed regarding their experiences of adjustment with a family caregiver present. After a pilot interview, a semi-structured interview was developed to explore caregivers’ experiences of support throughout the illness.

A subgroup of caregivers from the broader sample (*n* = 25) was selected to participate in this research. Purposive sampling was used to identify 12 caregivers with diverse characteristics likely to impact on perceptions of support, including tumor type, gender, age, and relationship to the person with brain tumor. The aim of purposive sampling is not to generalize findings to the larger population, but to select information rich-cases for study that provide an in-depth understanding of a topic (36). The primary sampling criterion was that participants were caring for an adult with a benign or malignant tumor, followed by selection on the basis of caregiver gender, age (<50, 50–60, >60 years) and relationship to the individual with brain tumor (married/*de facto* or parent). Although 12 caregivers were identified, 1 audio file was corrupted and therefore the data for participant 12 (the mother of 28-year-old woman with malignant brain tumor) could not be included in the study, resulting in a final sample of 11 caregivers. The demographic characteristics and pseudonyms of the caregiver participants are shown in Table 2.

**Table 2:** **Caregiver demographic characteristics and tumor type (note: the pseudonym and participant number are used to indicate caregivers’ gender, age, and relationship status to the person with brain tumor and the tumor type)**.

Caregiver characteristics		Tumor type			
		Grades I–II		Grades III–IV	
Relationship status		Married/*De facto*	Parent	Married/*De facto*	Parent
**Caregiver gender**	**Age**				
Male	<50	James (PT 4)			
	50–60	Sam (PT 9)		Barry (PT 1)	
	>60	Jim (PT 10)	William (PT 8)	Michael (PT 7)	
Female	<50			Wendy (PT 6)	PT 12
	50–60	Susan (PT 11)	Laura (PT 5)	Joanne (PT 2)	
	>60				Shelley (PT 3)

The 11 caregivers included 6 males and 5 females who were aged 33–79 years (*M* = 57.91, SD = 12.62). Six were married to the person with brain tumor, two were *de facto* partners and three were parents (mother = 2, father = 1). Six participants were caregivers of a person with a benign or low grade brain tumor and five were caregivers of someone with a malignant tumor. Caregivers’ level of education ranged from 9 to 18 years (*M* = 12.80, SD = 3.04). Two caregivers were working full-time, three were employed on a part-time/casual basis, one was a volunteer, one was unemployed, three were retired, and one caregiver did not provide this information.

Caregivers were supporting an individual with brain tumor who was between 9 months and 22 years post diagnosis (*M* = 5.88, SD = 6.30). All had undergone treatment involving surgery and either radiation, chemotherapy or both. The majority of individuals had one type of tumor; however, one person had three different tumors diagnosed at different time points (Wendy). Tumor types included Grade I or Grade II tumors (low grade astrocytoma = 2, meningioma = 3, colloid cyst = 1, unknown benign subtype = 1); and Grade III or Grade IV tumors (oligodendroglioma = 2, glioblastoma multiforme = 2, anaplastic astrocytoma = 1, unknown malignant subtype = 1).

### Measures

Caregivers completed the depression, anxiety and stress scales [DASS-42; (39)], caregiver strain index [CSI; (40)], and brief social support questionnaire [BSSQ; (19)] to provide descriptive information regarding their emotional wellbeing, level of demands experienced in their caregiving role and social support.

The DASS-42 is a 42 item questionnaire designed to assess symptoms of depression, anxiety, and stress. Each scale includes 14 questions and participants rate their responses from 0 “*did not apply to me at all*” to 3 “*applied to me very much*” with higher scores indicating increased levels of depression, anxiety, and stress-related symptoms.

The CSI is a 13 item measure of the degree of strain caregivers experience in their role. The yes/no items refer to physical, emotional, and financial strain, family, social, and work adjustments and demands on the caregiver’s time. A total score is calculated by summing the number of yes responses, with a score of 7 or higher indicating clinically elevated strain.

The BSSQ is a modified brief version of the social support questionnaire [SSQ; (41)]. Caregivers were asked to list up to nine people or services that have provided them with support since their loved one’s diagnosis. For each source of support, caregivers rated their level of satisfaction on a 6-point Likert scale ranging from 1 “*very dissatisfied*” to 6 “*very satisfied*.” These scores were averaged to derive a mean satisfaction with social support score, whereby higher scores reflected greater satisfaction with support.

### Data collection

In-depth semi-structured interviews were conducted in caregivers’ homes, with the exception of one caregiver (Laura) who elected to complete the interview over the telephone. Time was spent building rapport prior to the interview. The format and topics were designed to support caregivers to reflect back on the time when their family member was diagnosed with a brain tumor and to facilitate open dialog regarding their experiences of support, the impact on their relationship, and what they have learnt from their experience. Although the latter topic was not directly related to a research aim or question, it was considered as a positive topic on which to conclude the interview. Throughout the interview prompts were used to facilitate further discussion and topics of relevance to caregivers were explored in a responsive and flexible manner. The interview guide, questions, and example prompts were as follows.

#### Introduction to interview

Can I get you to think back to the time when (name of person with brain tumor) found out about the brain tumor. I would like to know about the different types of support received during that time.

1. Support
  ○ What were the different types of support received by (name of the person with brain tumor) following diagnosis? (Example prompts: during treatment, when leaving hospital, after hospital)
  ○ What type of support, if any, did you receive? (Example prompts: particular people at the hospital, medical, and nursing professionals, people in your own social network)
  ○ BSSQ: for each source of support identified, caregivers were asked to rate their satisfaction with the support received on a scale of 1 (extremely dissatisfied) to 6 (extremely satisfied).
2. Impact on relationships
  ○ What impact, if any, has the brain tumor had on your relationship with (name of the person with brain tumor)?
3. Lessons learned from experiences and insights to share with others in a similar situation
  ○ If you met someone today who just found out their relative has a brain tumor, would there be any advice you would give them, and if so, what would that be?

The two interviewers were females with an Honors degree in psychology, who were enrolled in a Masters or PhD in Clinical Psychology. Both received specific training in qualitative interviewing techniques (36). All interviews were audio-taped and the average recorded duration was 51 min (range = 27–88 min). Two caregivers, Shelley and Barry, chose to complete their interviews with their family member with brain tumor present.

### Procedure

The study was approved by a university human ethics committee prior to recruitment. All research procedures were conducted in accordance with the National Statement on Ethical Conduct in Human Research. People with brain tumor were initially approached by the coordinator of a brain tumor support service at the Cancer Council Queensland or the neuro-oncology nurse practitioner at a private neurosurgery clinic to discuss the study. If the person with brain tumor agreed to participate in the research, his or her caregiver was also approached. Researchers met with caregivers in their own homes (note: Laura was an exception as she preferred a telephone interview) and gained written informed consent. Caregivers participated in the interview first and then completed the questionnaires. The audio-recordings were transcribed verbatim prior to coding and thematic analysis. Sources of support identified by caregivers throughout the interview and comments regarding the benefits or effectiveness of support sources were tabulated using a frequency table during the transcription process. Throughout the transcription and analysis, a reflexive journal was kept by the researcher (Elizabeth Goadby) to record personal feelings and opinions to monitor any potential source of bias or influence on the findings (36).

### Data analysis

Descriptive statistics and frequency data were examined using IBM SPSS statistic software version 20. The qualitative analysis involved thematic analysis of the transcribed interviews based on the open, axial, and selective coding approach (42), as outlined in the following section. Although this analytic process is most commonly employed in grounded theory research, it is suitable for use in phenomenological studies as it facilitates in-depth understanding of subjective experience (43).

#### Open and axial coding

During these initial stages of coding, three caregiver transcripts were read through and a preliminary coding framework was developed. This framework highlighted a number of initial codes and categories relating to caregivers’ experiences of support and the impact of brain tumor on their relationship with the person with brain tumor. Using this preliminary coding framework, one transcript was coded separately by two authors (Tamara Ownsworth and Elizabeth Goadby). Consensus coding was conducted on the 39 paragraphs of the transcript, which yielded an agreement level of 74%. Through this initial process a number of changes were made to the coding framework and consensus coding was then completed on three additional transcripts (184 passages), which yielded an agreement level of 90%. In the instances where there were differences in coding, these were discussed to reach consensus on an appropriate category. The remaining eight transcripts were then read through and experiences that were consistent with this framework were identified. This process also highlighted new experiences not captured by the coding framework, and thus it was altered to incorporate different experiences through collaborative discussion between the researchers.

#### Selective coding

As the final stage of the coding process, selective coding involves in-depth reflection and discussion of the coding framework by the research team, to draw a higher level of abstraction and meaning from the data (42). The categories identified during the open and axial coding stages were examined and overarching themes around support and relationships were developed, along with a number of subthemes that were considered to best represent the experiences of the caregiver sample.

#### Strategies to enhance rigor

A number of strategies were utilized to enhance the rigor or trustworthiness of the findings and minimize the potential for researcher bias. These included keeping a reflexive journal, accounting for the “positionality” or background and preconceptions of the researcher (i.e., reflexivity) and consensus coding (36). A reflexive journal was kept by the researcher throughout the data transcription and analysis process to record personal feelings and opinions that emerged as the research proceeded, in order to monitor the influence of these experiences on the interpretation of the results. As described previously, consensus coding was utilized throughout each stage of data analysis. During the open and axial stages of coding, this process involved transcripts being independently coded and then discussed by two researchers. During the selective coding phase, in-depth and collaborative discussion occurred between members of the research team to facilitate scrutiny and clarity of the emerging themes (36).

The consensus coding process also helped to ensure that the researchers’ past experiences and preconceptions did not unduly influence the interpretation of the data. For example, in addition to her professional experience as a psychologist, the researcher primarily involved in the data analysis had personal experiences of caring for a relative with breast cancer and another relative with brain tumor. Her experiences as a caregiver were acknowledged and considered in the process of formulating the themes and subthemes to limit the potential for researcher bias (33–35).

## Results

### Caregiver wellbeing and perceptions of support

Caregivers were administered with the DASS and the CSI to provide information regarding their emotional wellbeing and the level of demands experienced in their caring role. Two caregivers (James and Laura) did not return the questionnaires despite multiple follow ups. According to the DASS, one caregiver (Susan) was experiencing a moderate level of anxiety (score = 10) and another caregiver (Wendy) was experiencing a moderate to severe level of stress (score = 25). Other caregivers were experiencing levels of depression, anxiety, and stress in the normal range on the DASS. The CSI indicated that most caregivers were experiencing non-clinical levels of strain (*M* = 3.55, SD = 2.29, range: 0–8). One caregiver, Joanne, reported a clinically elevated level of caregiver strain (score = 8/13).

Scores on the BSSQ indicated that although the number of sources of social support varied (range: 1–9/9), caregivers were typically satisfied with the support they received (*M* = 4.55, SD = 1.50). Table 3 provides the frequency data regarding the number of caregivers who identified different sources of support as well as their comments regarding the effectiveness or nature of support. Caregivers identified both informal (e.g., family and friends) and formal support from professionals in their health care team and community services.

**Table 3:** **Sources of support and caregiver comments on the nature of support (*N* = 9)**.

Source	*N*	Comments (no. of people providing comment)
Brain tumor support group	8	Sharing experiences with others (4)
		Provided information (3)
		Could not attend due to work conflicts (2)
International brain tumor website	4	Provided useful information (4)
Brain injury outreach service	1	Supportive (1)
Cancer support association	3	Provided information (2)
		Was unsure if helpline could help (1)
Hospital (generally)	2	Friendly staff (1)
		We never got a follow up call at home (1)
Oncologist	1	Nice manner of interacting (1)
Doctor (specialty not specified)	3	Lovely, explained it all (1)
		Knew who you were (1)
		Medical support, not emotional support (1)
Nursing staff	4	They knew our situation (1)
		Nice, but busy (1)
		Medical support rather than psychological (2)
		No follow up post discharge (1)
Social worker	4	Gave information about other services (1)
		Too busy to see us (1)
		No information about available services (2)
General practitioner	6	Emotional support (2)
		Provided reassurance (1)
		Provided information (3)
Neurosurgeon	6	Kind, caring, supportive (3)
		Provided information (1)
		No reassurance provided (2)
		Blunt, lacking in empathy, defensive (2)
Acupuncturist	1	Easy to talk to, supportive manner (1)
Psychologist	1	Supportive (1)
Government agency	2	Provided financial assistance (1)
		Friendly (1)
Health insurance	3	Helped to alleviate financial pressure (1)
		Provided financial assistance (2)
Cancer community (i.e., online, face-to-face contact)	3	Shared similar experiences (1)
		Shared information (2)
Family (children, partner, siblings, parents)	9	Emotional support (3)
		Practical support (e.g., making meals) (3)
		No emotional support (1)
		Infrequent support (1)
Friends	9	Supportive (3)
		Provided practical support (2)
		Did not know how to be supportive (2)
Neighbors	3	Supportive (1)
		Available for a chat (1)
		Provided practical support (1)
A person with brain tumor	1	Supportive (1)
Church	3	Supportive (2)
		No support offered (1)
Work colleagues	5	Supportive (2)
		Allowed time off (1)
		Financial support (1)

### Results of open, axial, and selective coding

The open and axial coding stages of the thematic analysis aimed to generate categories representing caregivers’ perceptions of support and the impact of the brain tumor on their relationship. Examples of key words and phrases highlighted during the open coding process included

- Friend: “We had a friend who was a good support for a little bit there”
- Knowledge: “The support that I was looking for was knowledge”
- Stronger: “It is made our relationship a lot stronger over time.”

During the axial coding process, key words and phrases with common meanings were grouped together to generate subcategories. For example, lending money, doing housework, and making meals were grouped together under the sub-category “Practical Support.” Practical support was grouped together with emotional support and information under the main category, “Types of Support.” A summary of the coding framework derived from the open and axial coding stages is presented in Table 4.

**Table 4:** **Summary of categories, subcategories, and example key words or phrases**.

Main category	Sub category	Example key words or phrases
Source of support	Health professionals	Nurse
		General practitioner
		Neurosurgeon
		Oncologist
		Social worker
		Psychologist
	Services	Brain tumor support group
		Cancer support association
		Health insurance provider
		Government (e.g., disability support)
		Brain injury outreach service
	Informal network	Family
		Friends
		Neighbors
		Work colleagues
		Cancer community
Type of support	Emotional	Someone to talk to
		Keeping in touch
		Being there when it mattered
		Sharing experiences
	Practical	Financial support
		Housework
		Made meals for us, childcare
	Information	Brain tumors
		Treatment
		Supporting someone with a tumor
Nature of support	Time frame	Not daily, but there when we needed it
		No support when we left hospital
		Only contacted every couple of months
	Manner of interacting	Warm, kind
		Gave hope
		Blunt
		Not empathetic
		Gave no reassurance
Factors impacting on support	Support being offered	Distance
		Short time between diagnosis and treatment
		People not knowing what to say
	Support being sought	Short time between diagnosis and treatment
		Shock after diagnosis
		We thought he would be ok
		Lack of knowledge about services
		Time constraints/work conflicts
		No services in our area
Impact on relationships	Changes in relationship	Made us closer
		Abuse and criticism all day
		Close before and still close now
	Changes in roles	Full-time parent
		Main breadwinner
		Increased household responsibilities
		Change in employment

Following in-depth reflection and discussion of the categories and subcategories by the research team, two overarching themes around support and relationships were identified. The first major theme was “Meanings of Support” and the second one was “Relationship Impacts.” The following section presents the major themes and subthemes related to support and relationships. Quotes from transcripts are used to illustrate caregivers’ experiences, and questionnaire data are drawn on when relevant to consider caregivers’ perceptions in the context of their levels of strain, emotional distress, and support.

#### Meanings of support

The first major theme that emerged from the data related to the different meanings of support for caregivers. It was not merely the presence or absence of support that appeared to influence caregivers’ perceptions, but rather the extent to which support met their expectations. The Meaning of Support theme was characterized by three subthemes, namely, intertwined and distinct support needs, varied expectations of support and factors influencing support expectations.

##### Intertwined and distinct caregiver support needs

When caregivers were specifically asked about their own support needs, some expressed that their support needs were closely intertwined with the needs of the person with brain tumor. For these caregivers, there was no distinction made between support perceived for themselves and that of the individual with brain tumor. For example, when Michael was asked if friends were supportive of him and his wife he replied: “*Yes, which are basically the same thing*.” These caregivers often used phrases like “*when we were diagnosed*” or “*when we went through treatment*,” highlighting their shared experiences of both the brain tumor and support.

Other caregivers reported feeling supported if their loved one was supported. Laura (mother): *The oncologist wasn’t personally supportive of me, I didn’t expect that, but by the fact that I knew she was so wonderful for my daughter that supported me*. When discussing support groups, which typically focused on the individual with brain tumor, some caregivers reported feeling supported if their loved one was receiving support and benefiting from this. Similarly, when asked to reflect on support he would have liked, James (partner) expressed: *Not really for myself – more for Lucy … for other people to support her*. These caregivers did not appear to expect support for themselves because their focus was on their family member. Although some caregivers acknowledged the general lack of support for caregivers, they were often unsure if they would have access this support had it been available. Sam (husband): *No one really worries about you (the caregiver); they worry about the person with the brain tumor. I’m not sure I needed it either*.

While most caregivers did not refer to seeking support specifically for themselves, one caregiver, Wendy (wife) identified her own need for psychological support. Distinct from other caregivers she reported significant changes in her husband’s personality: *I’ve actually started to admit to myself he’s not the person he used to be … you’ve lost that person you’ve married and you’ve got to deal with that*. Wendy reported moderate to severe levels of stress on the DASS and was seeking counseling to cope with her feelings of grief regarding changes in her husband.

One area in which caregivers often perceived their own distinct support needs was information. In particular, they wanted easy to understand information on what to expect when caring for someone with a brain tumor, including different types of brain tumor, treatment, and side effects. Barry (partner): *I wasn’t really seeking support, most of the support that I was looking for was knowledge*. Caregivers perceived that access to information would have helped them to adjust to their caregiver role. William (father): *Even if we had been aware of the support group and all the information available … that could have made our lives so much easier*.

##### Varied expectations of support

Caregivers held different expectations of support with respect to the time frame over which it was provided, the type or nature of support and the extent to which support should be offered to, or sought by the caregiver. In terms of the time frame, some caregivers had expected that support from family, friends, or professionals would continue throughout treatment and post-treatment. When asked about support following her daughter’s discharge from hospital, Joanne noted: *We never had any call back from them (hospital) … or a call at home to see if we got there, nothing*. Another caregiver reported ongoing support from family and professionals, but felt that the support was too infrequent. James (partner): *There were family members … and they kept in touch, but that was only every couple of months*. Other caregivers perceived that ongoing support even on a less frequent basis was supportive. Michael (husband): *Well, (Hospital) you know there was support there all the time …. Even now when we go in we still meet some of them*. Professionals were viewed as supportive when support was available as needed. For example, in discussing their GP, Sam (husband) noted: *He wasn’t a daily source of support, but when we had to go and talk to him he was excellent*.

Caregivers’ expectations of the type of support also varied. Most caregivers received a range of practical supports including financial assistance, house-keeping, childcare, and workplace flexibility. However, their perceptions of emotional support appeared to impact the most on their overall sense of feeling supported. For example, when reflecting on support from friends and family, Shelley (mother) expressed: *It’s the emotional support I think people need more than anything*. For Shelley, despite receiving support from multiple sources including friends, family, and professionals (BSSQ = 9/9 sources), a lack of emotional support from these sources appeared to influence her perception: *I don’t think we got very much support at all from anywhere*. Caregivers who received minimal or no emotional support typically reported low satisfaction with support, even if they received practical assistance. For example, James (partner) noted: *My parents have been there … but they’ve been more financial support when we really needed it, not emotional*.

Caregivers also perceived that emotional support from healthcare professionals was very important, particularly in their manner of interaction. When discussing his wife’s neurosurgeon Sam expressed: *His manner’s been very encouraging and very supportive and I would classify him as being a source of support*. Doctors with a kind and caring manner were perceived as providing emotional support even when giving bad news. Laura (mother): *She (neurosurgeon) had to give us some bad news some of the time … and you couldn’t ask for a better manner in her delivery of that bad news, or her support in what we were going through*. These two caregivers also described negative experiences with other medical professionals who were perceived as cold and clinical or offering little hope or reassurance. Sam (husband): *There was no hello, we walked into the room and he (neurosurgeon) looked up from his desk and said you’ve got a very large brain tumor and it is an eight hour operation*. Laura (mother): *(We asked) do you think she will live? and he very tersely told us well, you want to be grateful that we’re not dead now … from our point of view all we really wanted was a little bit of reassurance*.

Caregivers differed in their views on whether support should be offered to, or sought by them, which in turn influenced their support seeking behaviors. Some caregivers were very proactive in seeking the support they needed. Wendy (wife) lived in a remote community away from support and services and noted: *That’s something I had to strike out and find on my own*. These caregivers often used the Internet to access information about brain tumors and treatment and to share their own experiences with the online cancer community. Jim (husband), who utilized multiples website to search for information, noted: *I’ve taken to tumors like a hook to a fish. I just had to – I was hungry for information*.

Other caregivers had expected professionals and services to extend offers of support. When reflecting on support from the hospital Shelley (mother) expressed: *Nobody ever rang up and said oh your daughter’s got a brain tumor, how can I help you? You know I’m from the hospital what can I do?* Similarly, Joanne (wife) thought that services would contact her to provide information and support: *I mean no one has sent us a letter or gave us a phone call and said as soon as you had a cancer you want to come to this seminar*. For some caregivers, additional stressors had impacted their ability to seek support for themselves. For example, Shelley (mother) was also the caregiver of her husband who had terminal cancer.

##### Factors influencing expectations of support

Factors that appeared to influence caregivers’ expectations of support included the time frame between diagnosis and treatment, geographical distance, work commitments, lack of awareness about support available, and expectations about their family member’s prognosis.

Most caregivers recalled the shock they experienced in learning about the brain tumor. There was often a very short time frame between diagnosis and treatment. Barry (partner): *They looked at the CAT scan … and got her straight back in for an MRI and then it was within a week that the operation happened*. Caregivers advised that in the early stages following diagnosis they did not expect to receive nor seek support as they were more focused on treatment for their family member. Sam (husband): *That was a time I guess of great shock in terms of support no, you’re basically just dealing with the issue*. As an exception, Michael sought support from his church community and friends and family shortly after his wife’s diagnosis. He and his wife had previously experienced major health issues and were able to quickly mobilize the support that had helped them to cope in the past.

Several caregivers advised that they would have liked to receive more information about brain tumor once the initial shock had subsided. Sam (husband): *I guess we just wish that someone would have said to us right at the beginning here’s a very good guide, because when you have a brain tumor situation, oh you’re lost*. Susan (wife) noted: *I think that’s the time when some sort of support would be very helpful perhaps to a lot of families*. The range of support services available, and what to expect as a caregiver, were identified as important types of information helpful for caregivers to receive soon after diagnosis. Wendy (wife): *I think that’s one of the biggest problems with the services, it’s hard when you don’t know where to even begin … I did not know where to go really and I suppose that was half the problem of not getting help*. Some misconceptions about support services also posed a barrier to accessing support. For example, Susan perceived that people with benign tumor were unable to access support from a cancer support service. Other caregivers suggested the need for better publicity and marketing around services so people are more aware of the support they can access.

Practical issues such as time and distance and expectations about prognosis impacted caregivers’ expectations of support. In particular, caregivers’ work commitments reduced the amount of time they could spend looking for information on brain tumors or seeking options for support for themselves. James (partner): *I could have done with something myself but I was pretty busy working*. Those living a long distance away from family or friends had less expectation of support from their informal support network, and hence the person with brain tumor and caregiver had become more reliant on support from each other. Expectations of positive outcomes after treatment were perceived to impact support seeking for two caregivers. They were advised that their family members would regain their former functioning. Susan (wife) noted: *We did not know we needed any … all the indications were everything was going to be fine … and when everything is going to be fine you don’t need any help*.

In summary, the Meanings of Support theme highlighted variations in caregivers’ perceptions of their own support needs in relation to those of the person with brain tumor. However, there was a general consensus on the need for caregiver-specific information. Caregivers had different expectations regarding the timing and type of support received, which was influenced by various factors (e.g., work commitments, their family member’s prognosis).

#### Relationship impacts

From the caregivers’ perspective, the brain tumor was associated with three main relationship outcomes, namely, the experience of strengthened, maintained, or strained relations. Issues that appeared to influence these outcomes were mood and personality changes of the person with brain tumor, changes in caregiver roles and responsibilities and the quality of the relationship before the diagnosis. Social support was perceived to influence relationship outcomes to varying degrees.

##### Strengthened

Two caregivers noted that their relationship was strengthened by the experience of brain tumor. Susan noted that she and her husband now share more as a couple; *I think it has made us closer … I’m a lot more tuned into him than I was before*. She advised that there were no major changes in her husband’s personality and only minor changes in her household responsibilities, which they had coped with by re-structuring their home environment. She felt that the limited support from friends, family, and professionals had drawn them closer together: *We pulled together for the family because we’ve always lived away from our families*.

A second caregiver, Barry advised that he had been through his own health issues and his partner had cared for him. Their mutual experiences as caregivers had brought them closer as a couple: *I think things like that have happened with Sarah and me; we’ve grown very close together as soul mates*. Similar to Susan’s experience with her husband, there were no major changes to Sarah’s personality or abilities. Barry had also made small modifications to their home environment to make things easier for his partner; however, he did not perceive any major changes to his role or responsibilities within the household. Barry advised that his work was very supportive, allowing him to take days off and have remote access.

##### Maintained

Two parent caregivers (Laura and Shelley) reported no change in their relationship with their adult children. Both noted that they were close to their daughters before the tumor and continued to be close after diagnosis and treatment. Laura (mother): *We did then and still have a close relationship*. Neither caregiver reported changes in their daughter’s mood or personality, although both caregivers reported minor changes in their responsibilities, such as driving their daughters to appointments or spending more time looking after their grandchildren. These caregivers differed in their perceived support needs; Shelley, who also cared for her husband with terminal cancer, reported a need for more emotional support as a caregiver, whereas Laura felt emotionally support by her husband.

##### Strained

The remaining caregivers perceived that the brain tumor had placed varying levels of strain on their relationship with their family member. These caregivers often reported changes in their loved one’s mood and personality, such as irritability and frustration. Sam (husband): *That was hard to take, to cop the abuse and criticism all day long*. In one instance, the personality of the person with brain tumor was perceived to have dramatically changed. Wendy (wife): *I’ve had to grieve for the man I married even though I’ve still got him …. It’s hard because some days John is really almost like the old John and you could sort of, do you say something to him or not? Yeah that’s hard*.

Changes in roles and responsibilities, such as taking on more household chores and childcare, also contributed to relationship strain. James reflected on the changes in his life following his partner’s treatment: *I did not really have too much to do with kids. I was riding dirt bikes and having a good time out there and sort of being single, to looking after Lucy and having bubs and the whole tumor ordeal*. Many caregivers described taking on more of the decision making regarding finances as they become the main or sole breadwinner. For caregivers who took on additional roles and responsibilities, lack of support appeared to contribute to their experience of relationship strain. For example, Wendy reported few sources of support (3/9) and low satisfaction with the support received (score: 2/6).

For other caregivers, the quality of the relationship prior to the brain tumor and other pre-existing stressors impacted on their current relationship. For example, Sam discussed the loss of the family business and his wife’s earlier diagnosis of breast cancer as issues contributing to relationship strain prior to the brain tumor diagnosis. *I was finding I was getting a lot of abuse and this was months before the diagnosis*.

Overall, while most caregivers perceived that their family member’s brain tumor placed strain on their relationship, some perceived no change or a strengthening of their bond. Mood and personality changes, role demands and responsibilities, and the quality of the relationship prior to diagnosis appeared to influence relationship outcomes. Social support was not found to have a consistent influence on relationship functioning. In particular, a lack of support was perceived to bring one couple closer together, have minimal impact on some relationships, and place strain on others.

## Discussion

This study aimed to understand how caregivers perceive their own support needs and the impact of brain tumor on relationship functioning. The two main themes highlighted the different meaning of the concept of support and diverse ways in which brain tumor affects relationships. Caregivers’ perceptions of support were influenced by their expectations of the timing and type of support received. More generally, their sense of being supported was dependent upon their subjective understanding of what constitutes support (e.g., practical vs. emotional, short-term vs. long-term, frequent vs. infrequent).

Overall, caregivers tended to view their support needs as indistinguishable from, or secondary to those of the person with brain tumor. For some caregivers, no distinction was made between support for themselves and support for the person with brain tumor; hence, they typically felt supported if their loved one was receiving support. Other caregivers had not considered their own support needs because they viewed these as secondary to their family members’ treatment and support needs. Wasner et al. (31) also found that caregivers often prioritized the support needs of the individual with brain tumor over their own. The intertwined vs. distinct support needs subtheme represents a novel finding in the context of brain tumor.

Caregivers in the present study nevertheless expressed a desire to receive more information about brain tumor, treatment effects, what to expect in caring for someone with a brain tumor and options for support. Other research has found that access to information can reduce caregivers’ anxiety and frustration (20, 23, 44). For example, Cornwell et al. (23) reported that during the early stages of the illness (i.e., 2 weeks post discharge) caregivers were often not receptive to information offered to them because they felt overloaded with information and unable to process it, due to their worries about the person with brain tumor. In contrast, Schubart et al. (9) found that information seeking by caregivers was highest immediately following the diagnosis of brain tumor, and helped families to cope with changes in their loved one and the challenges of their role. These mixed findings concerning caregivers’ preferences for information soon after diagnosis may be related to the type of information and the way in which it is delivered.

Emotional support was also recognized as an important component of support. For some caregivers the provision of practical support alone (e.g., financial assistance) did not contribute to a sense of being supported if emotional support was perceived to be lacking. Other studies have similarly highlighted the importance of emotional support from caregivers’ informal support network, which can include support groups or meeting other people in a similar situation (3, 11, 44). Caregivers also stressed the importance of emotional support from health care professionals, as conveyed by a kind and reassuring manner of interaction. In research by Wideheim et al. (17), caregivers described encounters with health care professionals as positive when they patiently listened to and answered their questions. Conversely, health professionals who failed to provide reassurance or focused solely on physical care as opposed to emotional support were perceived as unsupportive (20).

Caregivers’ expectation of the timeframe over which support would be provided varied. For example, some caregivers perceived that formal and informal support was most important during the early stages of the brain tumor, whilst other caregivers expressed the desire for ongoing or long-term support. A similar issue found in previous research is that that while family and friends may initially offer practical and emotional support, this dwindles over time (3, 45). Such findings are concerning when considering that the stressors experienced by caregivers are often long-term and their support needs may potentially increase due to tumor recurrence and functional decline (17). Overall, the findings highlight the importance of seeking to understand individual caregiver’s expectations and preferences for support at different times throughout the illness.

The main issues found to impact on caregivers’ access to support in the present study were lack of awareness of available supports, time and work commitments, and expectations around support seeking. Although there is limited previous research on support seeking behaviors in caregivers of people with brain tumor, Arber et al. (5) found that caregivers who perceived a lack of support often developed their own strategies for accessing information to reduce the uncertainty and stress related to brain tumor. However, Schmer et al. (27) found that caregivers were less likely to seek or access support options (e.g., attend support groups) when they had high care demands placed upon them. Given the findings of previous research (19) that social support can buffer the effects of caring for someone with severe disability, there is a need for more accessible and flexible avenues of support for caregivers of people with brain tumor.

The experience of brain tumor was found to impact on relationships in different ways, including strengthened, maintained, and strained relations between the caregiver and person with brain tumor. The experience of major personality change, or the sense that the person is no longer who they were prior to the illness, and cognitive difficulties appeared to contribute to relationship strain. Other research suggests that the excessive strain placed on caregivers can contribute to a relationship breakdown (4, 9, 17). It is noteworthy that all caregivers in the present study were currently supporting the person with brain tumor, and therefore issues precipitating relationship breakdown could not be explored.

The complex cognitive and behavioral changes that distinguish brain tumor from other cancers have been documented in previous studies (1, 3, 28). In particular, behavioral problems have been found to contribute to caregivers distancing themselves from their loved one (28), and are associated with lower levels of caregiver mastery and greater depressive symptoms (46). The link between behavioral problems and relationship strain highlights the need for behavioral support interventions for this population. Encouragingly, Whiting and colleagues (47) have developed a multi-tiered intervention approach for managing cognitive and behavioral deficits after brain tumor. A single case study was used to evaluate a behavioral therapy and skills training intervention for reducing problem behaviors (e.g., excessive talking, lack of turn taking) displayed by the person with brain tumor. A second intervention focused on educating and training family members (*n* = 7) in a half-day workshop on managing challenging behavior. The third intervention for health professionals (*n* = 43) involved a one day workshop on psychoeducation and skills training for managing clients’ challenging behaviors. Each intervention was found to be effective in terms of decreasing target behaviors, increasing knowledge and use of strategies. These findings highlight the potential value of multi-level behavioral support interventions, although controlled trials are needed to determine their efficacy.

Despite the more common experience of relationship strain, other caregivers in the present study reported an ongoing closeness or strengthening of their bond with their family member. These caregivers did not report changes to the person’s personality or a major shift in their responsibilities. Rather, becoming caregivers for their spouse was perceived to have brought them closer as they shared more as a couple. Salander and Spetz (26) similarly found that couples with more open communication about the tumor developed a joint platform for coping. Conversely, when the individual with brain tumor would not share their experiences with their spouse and there was no shared understanding of the situation they were more likely drift apart. These observations suggest that couples therapy interventions may be beneficial for this population in addition to caregiver education and skills training (48). For example, Leboeuf (49) described a family centered approach used by a clinical nurse specialist to enhance communication and coping strategies of a couple in the context of malignant brain tumor.

The quality of the relationship prior to the brain tumor also appeared to influence relationship functioning in this study. Although this is a novel finding for brain tumor, previous cancer research found that caregivers who perceived a close relationship prior to diagnosis felt less burdened by caregiving and reported fewer depressive symptoms (50). Similarly, greater relationship satisfaction prior to the onset of dementia has been found to be associated with less burden and reactivity to cognitive and behavioral problems (51). Overall, the present findings suggest that a more cohesive relationship prior to the illness onset contributes to better dyadic adjustment. Couples with a pre-existing close bond may be able to draw upon their mutual knowledge of coping skills (e.g., communication and problem-solving) and commitment to support each other.

### Clinical implications

The finding that caregivers’ sense of being supported is subjectively construed highlights the importance of professionals seeking to understand their expectations of the timing and nature of support. Although a support needs assessment can be conducted by any health professional involved in the person’s care, some researchers have suggested that a designated staff member be allocated to provide tailored information and support throughout the illness (11). The integral support role of brain tumor care coordinators is being increasingly recognized (52); however, lack of funding is a key barrier to increasing the number of coordinator positions within the community. Pilot research suggests that a brain tumor specific question prompt list may help to reduce the unmet information needs of individuals with brain tumor (53), and this resource could potentially be extended to caregivers’ support needs.

As preferences concerning the timing and type of information received vary it would be beneficial to develop modular information kits that can be personalized to caregivers’ needs at different time points (e.g., shortly after diagnosis, before and after treatment, or following discharge). These modules could provide information about different types of brain tumor, treatment, side effects, care following discharge, long-term caregiver responsibilities, and support options. In the present study, online international forums and websites were perceived as valuable sources of support in addition to support services within people’s local area. Caregivers identified that living in remote areas or having work commitments posed barriers to accessing support services in person (e.g., attending support groups). Tele-health services offer considerable scope to overcome these barriers. In addition to video-conferencing with health professionals, web-cameras or teleconferencing may encourage caregivers to attend support groups remotely (54). Further, information seminars can be made available using audio-recordings and podcasts.

### Limitations

Although this study provides important insights into caregivers’ experiences of support and relationship, some limitations need to be considered. First, convenience sampling was used to recruit caregivers within a broader study focusing on the adjustment of individuals with brain tumor. While a purposive sampling strategy was used to select caregivers with diverse characteristics likely to influence perceptions of support, none of the caregivers had experienced a relationship breakdown with the person with brain tumor whereby they were no longer in a support role. Further, a larger sample size would have enabled exploration into the influence of caregiver gender on experience of support and the impact on relationships.

A prospective longitudinal study that monitors dyadic adjustment over time would enhance understanding of issues contributing to relationship strain and protective factors. A second related issue is that the interviews required caregivers to reflect back on their experiences of diagnosis and treatment which, for some, was over 20 years ago. It is possible that this approach affected caregivers’ recall of experiences relevant to their support and relationship functioning. As a third limitation, member checks were not used to enable caregivers to check their transcript after the interview or to more broadly verify that the key findings reflect their experiences (36). Finally, the present study was only concerned with caregivers’ perceptions of support and relationship outcomes. In future research it would be valuable to incorporate the perspectives of individuals with brain tumor on their caregivers’ support needs.

## Conclusion

This study identified variations in caregivers’ experiences of support and the effects of brain tumor on relationship functioning. The major finding concerning meanings of support underscores the value of seeking to understand what constitutes support for caregivers. Although caregivers varied in their expectations of the timing and type of support, most perceived the need for greater access to information and valued emotional support from professionals and their informal support network. Caregivers who perceived major changes in their family member, and had greater role adjustments typically experienced relationship strain. Overall, the findings highlight the importance of flexible and accessible support options and need for future research to evaluate caregiver support interventions.

## Author Contributions

All authors made a substantial contribution to the conception and design of the study, participant recruitment, data collection, and/or data analysis phases. Each author was involved in drafting the work or critically revising it for important intellectual content and gave final approval of the version to be published. All authors agree to be accountable for all aspects of the work in ensuring that questions related to the accuracy or integrity of any part of the work are appropriately investigated and resolved.

## Conflict of Interest Statement

The authors declare that the research was conducted in the absence of any commercial or financial relationships that could be construed as a potential conflict of interest.
